# Could Sodium-Glucose Co-Transporter-2 Inhibitors and Glucagon-like Peptide-1 Receptor Agonists Play a Role in Gout Treatment?

**DOI:** 10.3390/pharmaceutics17070865

**Published:** 2025-06-30

**Authors:** Dan Kaufmann, Naomi Schlesinger

**Affiliations:** Division of Rheumatology, Department of Medicine, Spencer Fox Eccles School of Medicine, University of Utah, Salt Lake City, UT 84132, USA; naomi.schlesinger@hsc.utah.edu

**Keywords:** gout, sodium-glucose co-transporter-2 inhibitors, glucagon-like peptide-1 receptor agonists, serum urate

## Abstract

Gout, a metabolic and autoinflammatory disease, is the most common form of inflammatory arthritis worldwide. Hyperuricemia may result in monosodium urate (MSU) crystals forming and depositing in joints and surrounding tissues, triggering an autoinflammatory response. Effective urate-lowering therapies, as well as anti-inflammatory medications, are used to treat gout. Over the past few decades, new antihyperglycemic drug classes with different modes of action have been added to treat hyperglycemia in type 2 diabetes mellitus (T2DM). Two of these drug classes, sodium–glucose co-transporter-2 (SGLT2) inhibitors and glucagon-like peptide-1 (GLP-1) receptor agonists (RAs), have reduced cardiovascular and renal events and mortality. Several clinical studies have demonstrated that SGLT2 inhibitors possess urate-lowering properties, which may be beneficial for treating gout patients, particularly those with comorbid T2DM. Regarding SGLT2 inhibitors, some researchers have suggested that their benefits are partly explained by their ability to reduce serum urate (SU) levels, probably through increased urinary uric acid excretion. The effect of GLP-1 RA on SU levels and urinary excretion of uric acid in humans is unclear. This paper reviews the mechanisms of action of SGLT2 inhibitors and GLP-1RA, both approved and in development. Additionally, it examines what is known about their structure–activity relationships, uricosuric effects, pharmacokinetic profiles, and adverse effects.

## 1. Introduction

Gout is the most common form of inflammatory arthritis, affecting approximately 5.1% of the US population. It is associated with elevated serum urate (SU) levels (>6.8 mg/dL), also known as hyperuricemia [1]. Type 2 diabetes mellitus (T2DM) and gout are comorbid metabolic disorders, and T2DM patients have an increased risk of gout, given the increased prevalence of a higher body mass index (BMI), hypertension, and chronic kidney disease (CKD) [2,3]. Therefore, glucose-lowering drugs that improve these risk factors can potentially reduce the risk of gout [4]. Hyperuricemia is independently associated with a greater risk of metabolic syndrome (MetS) and T2DM, potentially due to low-grade inflammation promoting diabetogenesis [5]. Current glucose-lowering drugs are limited mainly by their adverse event (AE) profiles, leading to the discovery and development of sodium–glucose co-transporter-2 (SGLT2) inhibitors and glucagon-like peptide-1 (GLP-1) receptor agonists (RA) [6].

## 2. SGLT2

**Figure 1:**
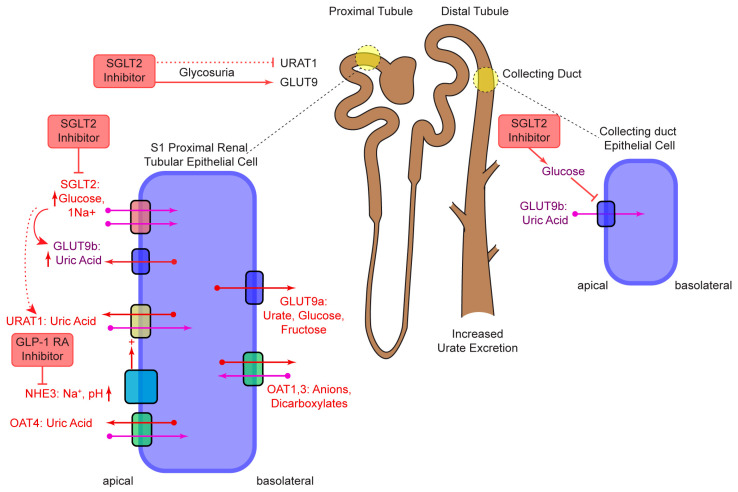
Hypothesized mechanisms of action of SGLT2 inhibitors and GLP-1RA. SLGT2 inhibition causes glycosuria which enhances urate secretion through GLUT9b in the apical membrane of the proximal tube (red arrow). Glycosuria may also inhibit urate reabsorption mediated through GLUT9b expressed in the collecting duct. SGLT2 inhibitors may also inhibit URAT1, as shown in animal models, but to a lesser extent than GLUT9 (dashed arrow). GLP-1RA inhibits Na^+^/H^+^-exchanger type 3 (NHE3) in the renal proximal tubule, thereby increasing natriuresis and alkalization of the urine, which can promote increased excretion of UA and reduce SU levels.

SGLTs are a family of membrane proteins expressed on the proximal renal tubules and the intestinal epithelium, responsible for active transport of glucose, vitamins, nucleotides, and amino acids coupled to sodium ions against their concentration gradient [7]. SGLT2 is a low-affinity sodium-glucose co-transporter with fourteen transmembrane domains; it is predominantly expressed in epithelial cells of the kidney’s proximal tubule and plays a major role in glucose reabsorption, preventing >90% glucose loss in urine (Figure 1) [8]. SGLT2 inhibitors lower blood glucose levels without altering insulin secretion and they exhibit uricosuric effects, making them a valuable therapeutic option [9].

**Figure 2:**
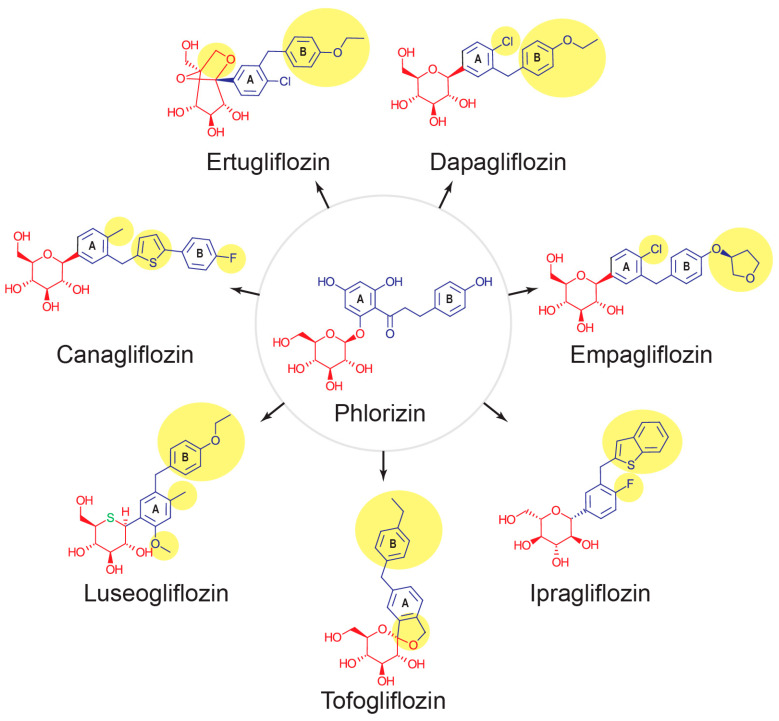
Chemical structures of phlorizin and clinically approved SGLT2 inhibitors. Each chemical structure has a glycon moiety (red) and an aglycon moiety (blue) with a proximal ring (A) and a distal ring (B). The yellow circles indicate the structural modifications responsible for increased activity and selectivity for the SGLT2 transporter.

Phlorizin (Figure 2) was the first SGLT2 inhibitor to be identified [10]. It is a naturally occurring phenolic O-glycoside found in plants, including the bark of apple trees [10]. Its chemical structure includes glycone and aglycone moieties, which consist of a central and peripheral phenyl ring (Figure 2). Phlorizin is a non-selective SGLT inhibitor that specifically and competitively inhibits SGLT2 and SGLT1 [11]. Despite its pharmacological activity, phlorizin has poor oral bioavailability, necessitating high doses to achieve therapeutic levels of SGLT2 inhibition, resulting in significant SGLT1 inhibition and gastrointestinal AEs [11]. Due to metabolic instability, non-selective inhibition of SGLT1, and interference with glucose transporter (GLUT)-mediated glucose transport, phlorizin is unsuitable for clinical use as a glucose-lowering drug [10,11]. Development of aryl-C-glucosides featuring meta-substituted benzyl groups restored SGLT2 inhibitory potency while retaining metabolic stability, a structural feature common to all approved SGLT2 inhibitors [10,11].

## 3. GLP-1

**Figure 3:**
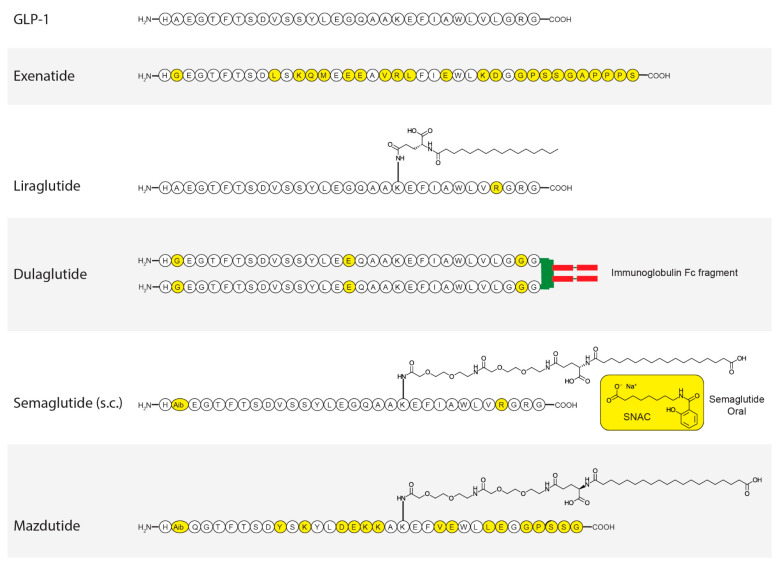
Structure of approved GLP-1RA and others in development. The yellow circles represent changes in amino acid compared with the original GLP-1 peptide.

GLP-1 (Figure 3) is a 30-amino acid incretin hormone with many effects, including glucose-dependent insulin secretion leading to lowering of glucose, delayed gastric emptying, increased natriuresis and diuresis, reduced food intake, modulation of pancreatic β-cell proliferation, attenuation of inflammation, and regulation of lipid metabolism [12]. The GLP-1 receptor (GLP-1R) is a seven-transmembrane Gαs-protein-coupled receptor member of the B1 subfamily that plays a key role in insulin and glucagon secretion [13]. Activation of GLP-1R stimulates insulin secretion via the incretin pathway, which is impaired in T2DM, making it a prime target for developing novel glucose-lowering drugs [14]. Consequently, GLP-1 mimetics were initially designed to enhance insulin secretion and manage T2DM [15]. GLP-1R is expressed in multiple human tissues, including the pancreas, intestine, lung, kidney, breast, brain, and cancerous tissues [16]. Within the central nervous system, GLP-1R activation reduces gastric motility and emptying, thereby limiting nutrient absorption and attenuating postprandial glucose spikes [15]. Additionally, GLP-1R is expressed in all four chambers of the heart, the sinoatrial node, and smooth arteriole muscle cells [17]. Consequently, GLP-1 binding to its receptor has been shown to reduce arterial blood pressure and increase cardiac output [18], contributing to overall cardiovascular (CV) health [15]. GLP-1 also induces natriuresis, potentially through suppression of angiotensin II [19,20] but independent of renal plasma flow and changes in glomerular filtration rate (GFR) [20]. These renal effects highlight GLP-1’s therapeutic potential in managing CKD in patients with T2DM. The mechanism of action underlying GLP-1 mimetics’ uricosuric effect is unclear. However, preclinical models have indicated that GLP-1 mimetics may inhibit Na^+^/H^+^-exchanger type 3 (NHE3) in the renal proximal tubule, resulting in increased excretion of uric acid (UA) and a reduction in SU levels.

Previous studies suggest that SGLT2 inhibitors and GLP-1 mimetics may lower SU levels and reduce gout risk. However, the extent of SU reduction and gout risk modulation varies among these drugs. This review examines approved SGLT2 inhibitors and GLP-1 mimetics and those in development, focusing on their chemical properties, pharmacological profiles, and potential therapeutic applications in gout management.

## 4. Approved SGLT2 Inhibitors, Serum Urate Lowering, and Their Possible Roles as Gout Treatments

### 4.1. Canagliflozin

Canagliflozin (Figure 2) was the first SGLT2 inhibitor approved in the USA and Canada, on 29 March 2013 [21]. It is marketed as Invokana as 100 mg/day oral tablets and can be increased to 300 mg/day [22]. Canagliflozin is a C-aryl glycoside and includes a substituted methyl in para-position on the proximal phenyl ring and a thiophene and a p-fluoro phenyl group as the distal phenyl group, increasing its SGLT2 inhibition profile and its selectivity over SGLT1 [6]. Canagliflozin is rapidly absorbed and has a time to peak drug concentration (Tmax) of 1 h, with a half-life of 16 h in healthy participants, and less than 1% is excreted unchanged in the urine [23]. It was associated with higher AEs than placebo in clinical trials, including genital mycotic infections, osmotic diuresis-related AEs, urinary tract infections, volume depletion-related AEs, and a transient reduction in GFR [24].

In a post hoc analysis of four phase III studies of T2DM patients, canagliflozin at 100 and 300 mg/day reduced SU levels by 13% (0.7 mg/dL) from baseline 5.3–5.4 mg/dL after 26 weeks [25]. Of 115 patients with baseline hyperuricemia (SU > 8 mg/dL), 23.5% and 32.4% achieved SU levels below 6 mg/dL after 26 weeks of treatment with 100 mg and 300 mg of canagliflozin, respectively [25]. That study did not include statistical comparisons; so, it is unclear whether canagliflozin’s uricosuric activity was significant.

A post hoc analysis of the Canagliflozin and Renal Events in DM with Established Nephropathy Clinical Evaluation trial (CREDENCE) showed that 100 mg/day canagliflozin (n = 153) significantly reduced SU levels after 52 weeks (−13.2 µmol/L, *p* < 0.05) compared to placebo (n = 224) [26]. The Canagliflozin CV Assessment Study (CANVAS) and CANVAS-Renal, two large randomized, double-blind, placebo-controlled trials, aimed to evaluate whether canagliflozin (100 and 300 mg/day) could reduce gout flares in T2DM patients with elevated risk of CVD [27]. A post hoc analysis (n = 10,142) indicated that canagliflozin reduced SU levels more than placebo (−23.3 µmol/L, −6.7% vs. placebo) after 6 weeks, with levels remaining stable at 3.6 years follow-up [27]. This effect was smaller than reported by Davies et al. (6.7% vs. 13% previously reported but larger than in the CREDENCE study (−13.2 µmol/L) [27].

The differences in uricosuria between these studies may be explained by the different populations with CVD (CANVAS study) and end-stage CKD (CREDENCE study) [26]. A meta-analysis indicated that canagliflozin and empagliflozin showed the highest reductions in SU levels compared with other SGLT2 inhibitors [28], although a more recent meta-analysis showed that empagliflozin had a higher uricosuric effect [29]. A recent meta-analysis of five randomized controlled trials (RCTs) involving 29,776 patients found that canagliflozin (100 and 300 mg/day) and dapagliflozin (10 mg) had a higher uricosuric effect and reduced risk of composite gout outcomes compared to other SGLT2 inhibitors [30]. In addition, canagliflozin reduced the risk of flares compared with placebo (4.1 vs. 6.6 patients with an event per 1000 patient-years, *p* < 0.0001), suggesting an anti-inflammatory effect beyond its uricosuria [27].

### 4.2. Empagliflozin

Empagliflozin (Figure 2) was approved by the FDA in May 2014 under the brand name “Jardiance” for the treatment of T2DM and in 2022 for adults with heart failure (HF) [21]. Empagliflozin can be given at 10 mg/day and increased to 25 mg/day as oral tablets. Empagliflozin is a C-glycosyl compound with a beta-glucosyl residue and a (4-chloro-3-{4-[(3S)-tetrahydrofuran-3-yloxy] benzyl}phenyl connected to the glucosyl anomeric center. The halogen substitution in the para-position of the phenyl ring is essential for inhibiting SGLT2 [6]. Empagliflozin is rapidly absorbed, and its Tmax is 1.33–3 h [31]. Its half-life has been reported as 5.6–13.1 h in single-dose studies and 10.3–18.8 h in multiple-dose studies. Mild to severe hepatic or renal impairments did not significantly alter the pharmacokinetics of empagliflozin [31].

The EMPA-REG OUTCOME trial studied the effects of empagliflozin (10 and 25 mg/day) in addition to standard care on CV morbidity and mortality in patients with T2DM at high CV risk (n = 7020) [32]. A post hoc analysis found that both empagliflozin doses (10 and 25 mg/day) reduced SU levels after 12 weeks, and the mean treatment difference from the placebo was −0.37 mg/dL (95% CI −0.42, −0.31 mg/dL). The reduction in SU levels was more significant when baseline SU exceeded 7.0 mg/dL and was sustained after week 12 [32]. It also significantly reduced the incidence of gout flares compared with placebo (21.6 vs. 14.1 events per 1000 patient-years, *p* = 0.001) at both doses [32]. Furthermore, empagliflozin improved all cardiorenal outcomes irrespective of pre-randomization SU level tertile [33]. The Empagliflozin Outcome Trial in Patients with Chronic HF and a Reduced Ejection Fraction (EF) (EMPEROR-Reduced) was a phase III randomized double-blind parallel group placebo-controlled trial that evaluated 3676 T2DM patients with HF for the effect of Empagliflozin (10 mg/day) on SU and worsening of HF [34]. In that study, 53% of patients had hyperuricemia, which was correlated with increased HF severity [34]. Empagliflozin significantly reduced SU levels after 4 weeks and remained lower throughout follow-up (−1.12 mg/dL vs. placebo, *p* < 0.0001). However, a more significant effect was seen in non-DM patients [34]. It also significantly reduced gout flares, gouty arthritis, or initiation of gout therapy by 32% (*p* = 0.004) [34]. In a randomized prospective intervention study, evaluated vs. the GLP-1RA liraglutide (1.8 mg/day) on 62 patients with T2DM, empagliflozin (25 mg/day) significantly decreased SU levels (−0.8 mg/dL, *p* = 0.003) after three months of treatment, while no change in SU was seen for liraglutide (0.6–1.8 mg/day) [35].

The EMPEROR-preserved trial was a phase III double-blind parallel-group, placebo-controlled event-driven trial that evaluated empagliflozin (10 mg/day) in 5988 HF patients with preserved EF [36]. Post hoc analysis on 5924 patients with chronic HF and preserved EF in the EMPEROR-Preserved trial evaluated SU reduction [37]. Hyperuricemia (6.35 ± 1.94 mg/dL for women and 6.86 ± 1.93 mg/dL for men) was prevalent in 49% of the patients and associated with advanced HF severity [37]. Empagliflozin (10 mg/day) significantly reduced SU levels within 4 weeks of therapy (−0.99 ± 0.03 mg/dL; *p* < 0.0001) and was stable throughout the treatment period (until week 172). Empagliflozin significantly reduced the risk of gout flares, gouty arthritis, and initiation of SU-lowering therapy by 38% (*p* < 0.0001). The SU reduction was more pronounced with higher baseline SU levels and was lowest among T2DM patients with severe CKD [37]. The larger uricosuric effect in non-DM patients may indicate that other mechanisms may drive empagliflozin’s uricosuric effect, rather than enhanced renal UA excretion [37]. The EMPA-KIDNEY trial was a randomized, double-blind, parallel group placebo-controlled phase III trial that evaluated 6609 patients with CKD (eGFR between 20 and 90 mL/min/1.73 m^2^) and baseline SU 431 ± 114 µmol/L receiving 10 mg/day empagliflozin vs. placebo [38]. Empagliflozin reduced average SU levels between groups (−25 µmol/L, *p* < 0.001), which were higher with higher eGFR and without DM [38]. SU levels were reduced after 2 months and remained stable up to 18 months [38]. However, the SU-lowering effect of empagliflozin was modest (relative reduction of 6%), lower than the SU-lowering achieved by xanthine oxidase inhibitors, and it did not reduce the risk of gout.

Compared with other SGLT2 inhibitors, empagliflozin had a greater uricosuric effect according to two meta-analyses [28,39]. However, another meta-analysis found luseogliflozin and dapagliflozin to be superior to empagliflozin, canagliflozin, and ipragliflozin [40]. The differences between empagliflozin’s uricosuric effects may have resulted from differences in population characteristics. Its greatest uricosuric effect was seen in patients with higher kidney function in the EMPEROR study, and was lower in CKD patients with T2DM on insulin at baseline, in the EMPA-KIDNEY trial [38].

### 4.3. Dapagliflozin

Dapagliflozin (Figure 2), marketed as Farxiga, received FDA approval on 8 January 2014. Farxiga is available in doses of 5 mg and 10 mg per day [21]. Dapagliflozin is a C-glycosyl with a D-glucose and a 4-chloro-3-(4-ethoxybenzyl) phenyl group. Similar to empagliflozin, it has a chlorine substitution on the proximal phenyl ring, and an ethoxy phenyl group, contributing to its increased potency [6]. Dapagliflozin is rapidly absorbed from the GI tract with an oral bioavailability of 78%. Its Tmax is 2 h, and it is predominantly eliminated via metabolism, including glucuronidation, dealkylation, and oxidation, with a half-life of 13 h [41].

The Dapagliflozin and Prevention of Adverse Outcomes in HF (DAPA-HF) study was a phase III placebo-controlled randomized trial that evaluated 4744 patients with HF and reduced EF with and without T2DM [42]. A follow-up study assessed the effect of dapagliflozin (10 mg/day) on SU levels in 3119 patients included in the DAPA-HF study with different SU levels at baseline (some of them already receiving urate-lowering therapies (ULTs)), divided into three tertiles (T1 SU < 5.4 mg/dL, T2 SU 5.4–6.7 mg/dL, T3 SU > 6.8 mg/dL) [43]. In that study, 31.6% of participants (29.4% men and 39.2% women) had hyperuricemia at baseline (>7.0 mg/dL for men, and >6.0 mg/dL for women), and 488 patients (10.3%) had a history of gout. Dapagliflozin significantly reduced SU compared with placebo in all SU tertiles (−0.84 mg/dL, *p* < 0.001) after 52 weeks, although greater SU reduction was seen in non-diabetic patients with lower glycosylated hemoglobin (HbA1c) [43]. Furthermore, dapagliflozin significantly reduced the initiation of ULT, and more patients achieved SU levels < 6.0 mg/dL after 12 months of treatment compared with placebo [43]. This change was more than double the reduction in SU seen with empagliflozin after 52 weeks in the EMPA-REG study, which recorded a decrease of −0.37 mg/dL. Additionally, a post hoc analysis was performed on patients in the DAPA-HF study, and on 6263 patients in the Evaluation to Improve the Lives of Patients with Preserved EF HF (DELIVER) study [44]. Dapagliflozin decreased the risk of worsening HF or CV death, irrespective of gout, and reduced the initiation of ULT by 57% and colchicine treatment by 46% compared with placebo in patients with or without a history of gout [44]. A small randomized placebo-controlled crossover study (n = 36, the QUARTZ study) evaluated SU levels after administrating a combination of dapagliflozin (10 mg/day) with febuxostat 80 mg/day and a URAT-1 inhibitor (verinurad 9 mg/day), compared with febuxostat and verinurad alone, in adults with asymptomatic hyperuricemia without a history of renal, hepatic, or gastrointestinal diseases [45]. Dapagliflozin reduced SU levels after 7 days < 6.0 mg/dL, showing a mean treatment difference of −62.3 µmol/L (23.5% reduction) compared with placebo (*p* < 0.05) and without increasing urinary excretion rates [45]. The combination of dapagliflozin, febuxostat, and verinurad was well tolerated and further enhanced SU levels compared with verinurad and febuxostat alone. However, reduced SU levels without an increase in UA excretion argue against the involvement of the GLUT-9 mechanism and may indicate that dapagliflozin reduces the production of UA by inhibiting purine metabolism or reducing cell death/turnover [45]. Another study that evaluated plasma metabolomics in patients with T2DM receiving 10 mg of dapagliflozin daily for 12 weeks found a reduction in three metabolites related to xanthine metabolism. This reduction may enhance the effect of dapagliflozin in lowering purine catabolism and, consequently, lowering SU [46].

### 4.4. Luseogliflosin

Luseogliflozin (Figure 2) was approved in Japan in 2014 and is marketed under the brand name Lusefi [21]. The recommended oral dose is 2.5 mg/day, which can be increased to 5 mg/day [21]. Unlike previously described SGLT2 inhibitors, luseogliflozin has an S-pyranose instead of an O-pyranose ring. The addition of a methoxy group on position 6 of the proximal phenyl ring increases its efficacy [6]. The addition of an ethoxy phenyl ring (similar to dapagliflozin) increases its selectivity for SGLT2 [6]. Luseogliflozin is quickly absorbed from the GI tract and reaches Tmax after 0.6–2.2 h. It has a half-life of 9–13.8 h and is predominantly eliminated via metabolism [47].

Luseogliflozin was evaluated for its uricosuric efficacy after single and multiple doses in 57 and 24 healthy subjects, respectively. It was found to decrease SU levels in a dose-dependent manner after a single dose (1–25 mg) and after multiple doses after 7 days compared with baseline [48]. A phase II randomized placebo-controlled double-blind study evaluating 12-week administration of 0.5, 2.5, and 5 mg/day luseogliflozin in 232 patients with T2DM (Japic) found significantly reduced SU levels compared with placebo at 2.5 (−0.63 mg/dL, *p* < 0.05) and 5 mg/day (−0.57 mg/dL, *p* < 0.05) but not 0.5 mg/day (−0.43 mg/dL, *p* > 0.05) [49]. A follow-up randomized double-blind placebo-controlled parallel-group comparative phase III study (Japic) of 148 Japanese patients with T2DM evaluated 24-week administration of 2.5 mg/day luseogliflozin vs. placebo and found a significant SU-lowering effect compared with the placebo (−0.34 mg/dL, *p* < 0.001) [50]. Pooled analysis of 52-week administration of 2.5 mg/day luseogliflozin in T2DM patients divided the participants into five groups based on their eGFR; it was found that luseogliflozin significantly reduced SU levels after 52 weeks (between −0.35 mg/dL to −0.49 mg/dL, *p* < 0.001) in T2DM patients with normal to moderate CKD (eGFR ≥ 45 mL/min/1.73 m^2^), but not in patients with moderate to severe CKD (eGFR ≥ 30 to < 45 mL/min/1.73 m^2^) [51]. Thus, luseogliflozin’s efficacy was reduced with worsening CKD, but it was well tolerated by all patients [51]. Notably, luseogliflozin significantly decreased SU levels in patients with higher baseline BMI (*p* < 0.001) [52].

### 4.5. Ipragliflozin

Ipragliflozin (Figure 2) was approved in Japan in 2014 under the brand name Suglat but has not been approved in the USA or European countries [21]. The recommended dose is 50 mg/day and can be increased to 100 mg/day [21]. It is a C-glycosyl compound with a substituted fluoro group in the para position on the proximal ring (unlike chloro groups in empagliflozin and dapagliflozin), which is essential for its glucose-lowering properties [6]. Ipragliflozin is rapidly absorbed, with a Tmax of 1 h; its half-life is 10–15 h, and it is mainly eliminated by metabolism, while only 1% is excreted unchanged in the urine [47].

A phase II double-blind multicenter placebo-controlled dose-response study evaluated the efficacy and safety of 12 weeks of ipragliflozin (12.5, 25, 50, or 100 mg/day) in 360 Japanese patients with T2DM [53]. Reductions in SU levels were not significant compared with placebo [53], whereas in the ASSIGN-K multicenter prospective intervention study (University Hospital Medical Information Network Clinical Trials Registry), 50 mg/day ipragliflozin for 24 weeks significantly reduced SU levels in 367 T2DM patients at week 4 compared to the baseline (−0.4 ± 0.9 mg/dL), and for up to 24 weeks after ipragliflozin treatment (*p* < 0.001) [54]. The differences between these studies with regard to ipragliflozin’s uricosuric activity may have resulted from the group comparison in the 12-week study, which did not reach statistical significance vs. intragroup comparison in the ASSIGN-K study. Another 12-week randomized, open-label, active-controlled trial evaluated 50 mg/day ipragliflozin vs. placebo in 30 patients with inadequately controlled T2DM and found that it significantly lowered SU levels after 12 weeks (−44.0 µmol/L, *p* = 0.003) compared to the control [55].

One meta-analysis found ipragliflozin to have the lowest SU-lowering efficacy compared with other SGLT2 inhibitors. In contrast, another meta-analysis found that ipragliflozin was better than canagliflozin but not empagliflozin, dapagliflozin, and tofogliflozin [39,40].

### 4.6. Tofogliflozin

Tofogliflozin (Figure 2) was approved in Japan in March 2014, and its brand name is Apleway [21]. The recommended dose is 20 mg/day taken before or after food [21]. Unlike other SGLT2 inhibitors, tofogliflozin includes cyclization of the furan ring connecting the proximal phenyl ring to the glycone moiety, which increases its selectivity for SGLT2. In addition, it has a lipophilic electron-donating group at the para position, which is essential for its glucose-lowering properties [6]. Tofogliflozin has high bioavailability (97%) compared with other SGLT2 inhibitors. It has a Tmax of 2 h, a half-life of 5–6 h, and 16% is excreted unchanged in the urine [47]. In a phase IV multicenter, double-blind, placebo-controlled trial evaluating the effects of 16 weeks of 20 mg/day tofogliflozin vs. placebo (and 36 weeks open label extension) in 211 T2DM patients with inadequate glycemic control, tofogliflozin significantly reduced SU levels after 16 weeks (−0.18 mg/dL) compared to placebo (*p* = 0.0062) [56]. Another randomized, placebo-controlled, double-blind, multicenter parallel-group trial combined phases II and III (Japic), and evaluated 229 Japanese patients with T2DM. Patients were randomized to three doses of tofogliflozin (10, 20, and 40 mg/day) administered for 24 weeks and compared to placebo. Both 10 and 20 mg/day but not 40 mg/day tofogliflozin significantly reduced SU levels compared to the baseline and placebo (−0.3 mg/dL and −0.33 mg/dL for the 10 and 20 mg/day, respectively, *p* < 0.01 compared to baseline and placebo) [57].

### 4.7. Ertugliflozin

Ertugliflozin (Figure 2) was approved by the FDA in December 2017 under the brand name Steglatro [58]. Ertugliflozin was developed by modifying the glycon moiety and introducing the first spirocyclic sugar with good potency, increased selectivity and activity for SGLT2, and good physicochemistry and oral exposure [6,11]. Ertugliflozin is 100% bioavailable and is rapidly absorbed, with a Tmax of 1 h. It is eliminated by both metabolism and urinary excretion (51%) [47].

The VERTIS CV trial was a multicenter, double-blind, placebo-controlled phase III trial that evaluated the effects of 5 and 15 mg/day ertugliflozin compared with placebo on CV and kidney function in 8246 patients with T2DM and atherosclerotic CVD [59]. Ertugliflozin reduced baseline SU levels (−0.19 mg/dL for the pooled ertugliflozin groups, *p* < 0.001) by week 6, for up to 260 weeks. Ertugliflozin reduced the incidence of gout flares, which was identified by searching the adverse event database (8.9 per 1000 person years incident rate), compared to placebo (11.8 per 1000 person years incident rate). The hazard ratio for composite gout-related outcomes (gout onset, initiation of ULT in participants without a history of gout) was 0.76 compared to placebo (*p* = 0.0052) [59].

## 5. SGLT2 Inhibitors in the Pipeline

We did not find any studies on hyperuricemia or gout regarding the new SGLT2 inhibitors in the pipeline: bexagliflozin, remogliflozin carbonate, and sotagliflozin.

Table 1 summarizes the clinical data for SGLT2 inhibitors.

## 6. Approved GLP-1RAs, Serum Urate Lowering, and Their Possible Roles as Gout Treatments

GLP-1RAs have shown promising results in glycemic control and weight loss [60]. Hyperuricemia is more prevalent among individuals with DM, and the presence of DM increases the risk of developing hyperuricemia [60]. GLP-1RAs are divided into two categories: short-acting and long-acting drugs. Short-acting drugs, such as exenatide and lixisenatide, work by delaying gastric emptying and reducing glucose spikes after meals. In contrast, long-acting drugs, including liraglutide, dulaglutide, and semaglutide, maintain GLP-1 receptor activation over an extended period. This leads to decreased fasting blood glucose levels and more significant improvements in HbA1c levels. However, long-acting GLP-1 mimetics have a more limited effect on gastric motility and are, therefore, less effective at controlling postprandial hyperglycemia compared with short-acting drugs [15].

### 6.1. Exenatide

Exenatide (Figure 3) is a short-acting GLP-1RA that in April 2005 was approved for T2DM as a twice-daily preparation [15]. It is a 39-amino-acid peptide and a synthetic form of exendin-4, naturally occurring in the salivary secretion of the lizard Heloderma suspectum [61]. In animal models of T2MD, exendin-4 improved glycemic control through a combination of mechanisms, including glucose-dependent insulin secretion, suppression of glucagon secretion, improvement of beta cell mass, slowing of gastric emptying, reduction of food intake, and modulation of insulin-sensitivity in peripheral tissues [61]. An important difference between exenatide and GLP-1 is in the presence of Gly^2^ instead of Ala^8^ (in GLP-1) in their N-terminal, which results in protection from dipeptidyl peptidase IV (DPP-IV) degradation, thus increasing exenatide’s half-life [62]. Exenatide can be administered subcutaneously (SC) and has a 2.4 h half-life. However, introducing Gly^2^ also reduced its GLP-1R binding.

In a non-blinded, placebo-controlled study, 9 healthy overweight participants had exenatide (10 µg) infused for 150 min after 90 min of a placebo infusion [63]. A post hoc analysis showed that baseline SU levels significantly increased upon receipt of exenatide [64]. Exenatide did not affect UA’s fractional urinary excretion but increased UA’s absolute urinary excretion (*p* = 0.02). Another randomized, double-blind, placebo-controlled parallel group study examined the effects of exenatide infusion (10 µg) in 52 T2DM patients [65]. Post hoc analysis showed that baseline SU levels did not change following exenatide infusion compared with placebo. However, fractional and absolute urine excretion of UA increased compared with placebo and were correlated to urine pH but not to SU [64]. It may be that the short duration of exenatide administration explains the increase in excretion of UA, but the lack of SU lowering. The authors hypothesized that the immediate rise in excreted UA could be explained by exenatide’s inhibition of Na^+^/H^+^-exchanger type-3 (NHE3) in the renal proximal tubule, thereby increasing natriuresis and alkalization of the urine, which can promote increased UA excretion [64]. However, a decrease in expression of NHE3 augmented urate reabsorption in the renal proximal tubule in a rat model, indicating that NHE3 inhibition may not be the sole responsible uricosuric mechanism of exenatide or GLP-1RA [66]. A prospective randomized clinical trial involving 44 obese individuals with T2DM evaluated the effect of exenatide (5 µg twice daily for 4 weeks, followed by 10 µg twice daily if tolerated until week 26); no significant differences were seen in SU levels between exenatide or placebo or compared with the baseline [67].

### 6.2. Dulaglutide

Dulaglutide (Figure 3) was approved by the FDA in 2014 for once-weekly SC administration, under the brand name Trulicity [68]. Dulaglutide is a DPP-IV-protected GLP-1 analogue covalently linked to a human IgG4-Fc heavy chain by a small peptide linker [68]. Dulaglutide reaches its Tmax after 48 h, has a half-life of approximately 5 days, and is degraded into its amino acid components instead of being excreted in the urine or metabolized by cytochrome P450 enzymes [69].

A study evaluated dulaglutide 1.5 mg/week in 20 patients with T2DM with an insufficiently controlled glycemic index previously treated with 10 mg/day empagliflozin for at least 3 months. Baseline SU was 6.9 ± 1.4 mg/dL, which was significantly reduced after 3–6 months of treatment by −0.12 ± 0.24 (*p* < 0.05) [70]. In two open-label studies, no changes in SU levels were seen after 24 weeks [71,72].

### 6.3. Semaglutide

Semaglutide (Figure 3) is a long-acting GLP-1RA, first approved in the USA in 2017 (Ozempic) [15]. It was also approved for treatment of obesity in adults in 2021 under the brand name “Wegovy”. Its development was inspired by liraglutide, aiming to increase its half-life but retain liraglutide’s low immunogenic profile [73]. Its structure resembles GLP-1 with C-18 carboxylated fatty acid coupled to Lys^26^ via a hydrophilic linker, which enhances its affinity to albumin compared with liraglutide and increases its half-life to ~1 week in humans [73,74]. Semaglutide also substituted Aib^8^ and Arg^34^ instead of Ala^8^ and Leu^34^ in GLP-1. It can be administered SC at 0.5–1.0 mg once weekly, or orally (Rebelsus is the brand name of the oral tablet), in addition to sodium *N*-[8-(2-hydroxybenzoyl)-aminocaprylate] (SNAC) at 7–14 mg/day [73].

T2DM patients showed dose-dependent weight loss, and 40% of patients lost more than 10% of their weight [75]. A retrospective study evaluated the effects of semaglutide in 50 patients with T2DM [76]. Seven of them were naïve to semaglutide, and the rest switched from other GLP-1RA drugs. Of these, 24 had received dulaglutide, 18 had been taking liraglutide, and 1 had been prescribed exenatide). Semaglutide was initiated at 0.25 mg once weekly and increased to 0.5 mg once weekly after 4 weeks. The dose was increased to 1 mg once weekly in case of low efficacy. Blood and urine samples were collected after 3 and 6 months following the initiation of semaglutide. Baseline SU was 4.9 ± 1.03 mg/dL and was significantly reduced to 4.59 ± 1.03 (*p* < 0.01) [76]. Another retrospective study evaluated the effects of semaglutide on weight-loss body composition and muscle strength in 43 Chinese participants with obesity who received 24 weeks of semaglutide treatment (0.25 mg/week increased every two weeks up to 1.0 mg/week for 24 weeks) and lifestyle intervention changes (eating a low-balanced diet and 150 weekly physical activity) [77]. Baseline SU (400 µmol/L ± 98.0) was significantly reduced at week 24 after semaglutide administration (346.6 µmol/L ± 66.5, *p* < 0.01) [77]. However, a prospective trial in 20 T2DM patients evaluated the effects of three-month administration of 10 mg/day empagliflozide (n = 10) vs. semaglutide (0.25 mg/week SC increased to 0.5 mg/week on week 5 and 1 mg/week on week 9, n = 10) on inflammatory markers and glycemic control and found that semaglutide did not lower the SU level; however, empagliflozin significantly lowered SU levels after 3 months (*p* < 0.05) [78]. No studies so far have evaluated its effect on gout outcomes.

### 6.4. Liraglutide

Liraglutide (Figure 3) is a long-acting GLP-1RA, approved by the FDA in January 2010 for T2DM treatment (Victoza) as an injectable formulation. It is currently also approved for treatment of obesity (Saxenda) in adults (2014) and adolescents (2020) as well as for T2DM [15]. Given the short half-life of native GLP-1, development approaches for GLP-1RAs involved increasing their half-lives. Binding to plasma albumin is one approach used to extend the half-lives of GLP-1RAs, having been applied for the development of liraglutide and then for semaglutide [79]. The development of liraglutide followed the attachment of fatty-acid side chains to help bind to albumin and thus improve the half-life of the new compound. Liraglutide is a palmitate fatty acid connected via a γGlu linker to Lys^26^, which increases its half-life and thus provides a once-daily SC analogue of GLP-1. Compared with GLP-1, liraglutide has an Arg^34^ substitution to prevent fatty acid coupling [80] and an analogue that can be used in a semi-recombinant process [79]. Liraglutide has a half-life of 8–10 h after intravenous and 13–15 h after SC administration, respectively [74], and can be administered up to 1.8 mg/day for T2DM and up to 3.0 mg/day for obesity [79].

A retrospective study evaluated the efficacy of 0.6 or 1.2 mg/day liraglutide for 12 weeks in 46 non-diabetic obese patients with MetS [81]. All patients had hyperuricemia at baseline (7.2–7.5 mg/dL) and after 12 weeks, both doses significantly lowered SU levels (−1.4 mg/dL, *p* = 0.002 and −0.4 mg/dL, *p* = 0.041 for the 0.6 and 1.2 mg/day doses, respectively) [81]. Another retrospective study evaluated weight reduction, waist circumference, and HbA1c in 54 obese patients with T2DM who started treatment with liraglutide [82]. SU levels were also significantly reduced after 4–12 months of liraglutide administration (−0.69 mg/dL, *p* = 0.04); however, none of the participants had hyperuricemia at baseline [82]. A longitudinal study evaluating T2DM patients (n = 139) with renal impairment divided patients into three groups receiving three different antidiabetic drugs: liraglutide (0.9 mg/day), sitagliptin (50 mg/day), and linagliptin (5 mg/day) [83]. In total, 45 patients received 0.9 mg/day liraglutide (the approved upper dose in Japan) for 48 months, and 32 had their SU levels evaluated. The mean SU level at baseline was 6.7 ± 2.5 mg/dL, but no significant changes in SU were seen after 12, 24, 36, or 48 months, [83]. Another study compared an SGLT2 inhibitor and GLP-1RA administration for 36 months in 188 patients with T2DM and renal impairment (eGFR ≥30 mL/min/1.73 m^2^ and < 60 mL/min/1.73 m^2^, albuminuria < 1000 mg/gCr) [84]. The GLP-1RA group received 0.9 mg/day liraglutide, and the SGLT2 inhibitor group received either 5 mg/day dapagliflozin or 10 mg/day empagliflozin [84]. The liraglutide (n = 72) and empagliflozin/dapagliflozin (n = 84) groups significantly reduced SU levels. Liraglutide had a −0.5 mg/dL after 36 months (from 6.2 ± 1.7 at baseline to 5.7 ± 1.5, *p* < 0.05), and the empagliflozin/dapagliflozin group had a −0.8 mg/dL (from 6.0 ± 1.6 mg/L to 5.2 ± 1.4 mg/dL, *p* < 0.05). The empagliflozin/dapagliflozin group had a significantly lower SU than the liraglutide group (*p* < 0.05) [84]. Another open-label parallel-group randomized controlled trial compared 24-week treatment of liraglutide (0.9 mg/day) and empagliflozin (10 mg/day) combined with insulin therapy in T2DM patients who had never received a GLP-1RA, SGLT2 inhibitors, nor DPP-IV inhibitor treatment for at least 8 weeks before the study [85]. Both groups had SU levels < 6.0 mg/dL, and neither the liraglutide nor the empagliflozin groups showed reduced SU levels after 24 weeks [85]. Another prospective, multicenter observational study evaluated the effects of 12 weeks of liraglutide (starting at 0.3 mg/day up to 0.9 mg/day in week 12) on T2DM patients (n = 151) [86]. After 12 weeks, the SU levels were significantly reduced by −0.2 mg/dL (baseline 5.6 ± 1.5 mg/dL to 5.4 ± 1.4, *p* = 0.025); however, none of the patients had hyperuricemia at baseline [86]. A non-randomized controlled interventional study in obese T2DM patients (n = 15) evaluated the effects of 3-month liraglutide treatment (0.6 mg/day to 1.2 mg/day after 2 weeks) on parameters of insulin resistance and plasma adropin concentrations, indicating that liraglutide did not change SU levels from the baseline [87]. A parallel group, randomized, double-blind study involving 62 participants with inadequately controlled T2DM evaluated 5-week treatment with liraglutide (0.6 mg/day titrated to 1.2 mg/day after 1 week n = 31) vs. placebo (n = 31) in terms of effects on ambulatory blood pressure and heart rate [88]. No changes in baseline SU levels were seen after liraglutide treatment after 5 weeks, which was similar to the placebo group [88]. The LOSE-IT trial, a randomized double-blind placebo-controlled parallel group study including obese individuals with knee osteoarthrosis, offered an 8-week intensive Cambridge weight plan intervention followed by randomization to receive either 3 mg/day liraglutide or placebo for 52 weeks [89]. SU levels in the initial diet group were reduced by −0.21 mg/dL (n = 155), followed by an additional reduction in SU levels in the liraglutide group (n = 69, −0.48 mg/dL), significantly more than the placebo group (n = 65, −0.07 mg/dL) [89].

## 7. GLP-1RAs in the Pipeline

### 7.1. Mazdutide

Mazdutide (also known as IBI 362 or LY3305677, Figure 3) is a GLP-1RA and glucagon receptor dual agonist used as a once-weekly treatment for T2DM and obesity [90]. Mazdutide achieves Tmax after approximately 7 h, and its half-life is between 7.3 days and 44.8 days [91].

It has completed four phase III clinical trials in overweight or obese patients (the GLORY-1, GLORY-2, DREAMS-1 and DREAMS-2). The GLORY-1 trial was a randomized, double-blind, placebo-controlled trial on 610 overweight or obese adults [92]. Mazdutide was administered at 4 mg/week (n = 203) and 6 mg/week (n = 202) for 48 weeks and compared with placebo (n = 205). Mazdutide significantly reduced SU levels at week 48 (−36.70 µmol/L ± 4.744, *p* < 0.0001). A randomized, double-blind, placebo-controlled phase 2 study evaluated 24-week administration of mazdutide (3, 4.5, and 6 mg/week) in overweight and obese adults (n = 248) [90]. At week 24, mazdutide significantly reduced SU levels compared with placebo (−81.9 ± 10.1 µmol/L (*p* = 0.0003), −88.2 ± 9.5 µmol/L (*p* < 0.0001), and −105.9 ± 9.7 µmol/L (*p* < 0.0001), for 3, 4.5, and 6 mg/week doses, respectively) [90]. A phase 1b randomized, placebo-controlled, dose-escalation, multiple ascending doses trial in twelve overweight or obese individuals evaluated three mazdutide doses (up to 3, 4.5 and 6 mg/week) for 12 weeks [93]. Only the 4.5 and 6 mg/week doses differed significantly from the placebo [93]. Another randomized, placebo-controlled, multiple-ascending dose phase 1b study evaluated once-weekly mazdutide or placebo on Chinese overweight or obese adults (n = 24) [91]. Mazdutide was administered for 12 weeks at target dose of 9 mg/week (3 mg weeks 1–4, 6 mg weeks 5–8, 9 mg weeks 9–12) and for 16 weeks at target dose of 10 mg/week (2.5 mg weeks 1–4, 5 mg weeks 5–8, 7.5 mg weeks 9–12, 10 mg weeks 13–16) [91]. Both doses reduced SU levels after 12 weeks (for the 9 mg/week dose) and 16 weeks (for the 10 mg/week dose); however, no statistical analysis was performed [91].

### 7.2. Tirzepatide

Tirzepatide is a dual GLP-1/glucose-dependent insulinotropic polypeptide (GIP) receptor agonist. Most clinical trials for tirzepatide have focused on weight, HbA1c, cardiovascular, and renal outcomes, rather than gout or SU. Other GLP-1/GIP receptor agonists in development, such as Retatrutide (LY3437943) and MAR709, also currently lack published data related to gout or hyperuricemia.

Table 2 summarizes the clinical data for GLP-1RA.

## 8. Discussion

In conclusion, SGLT2 inhibitors have uricosuric effects. However, the overall percent reduction in SU is lower than that seen with conventional ULT, and the relative decrease in gout flares is similar, suggesting that they have effects other than their uricosuric impact [49,58]. Furthermore, unlike conventional ULT, SGLT2 inhibitors do not induce flares at the beginning of treatment [58,90]. The downregulation of the pentose phosphate pathway may partly clarify how SGLT2 inhibitors function [91].

Emerging evidence indicates the greater therapeutic potential of SGLT2 inhibitors compared with GLP-1 mimetics for gout treatment. In a Danish study of 11,047 participants, initiation of SGLT2 inhibitors was associated with a lower incidence of gout (4.7 per 1000 person-years) than initiation of GLP-1RA (7.0 events per 1000 person-years) [94]. A similar result was obtained in 295,907 adults with T2DM, with a lower incidence of gout after initiation of SGLT-2 inhibitor therapy than GLP-1RA therapy (difference rate: 2.9 per 1000 person-years) [95]. In a large study on T2DM patients treated with metformin or insulin, which used an international federated database, a statistically significant decrease in the incidence of gout was observed 5 years after the introduction of SGLT2 inhibitors, but not with GLP-1 mimetics [96]. A recent meta-analysis evaluating 22 clinical trials indicated that SLGT2 inhibitors can reduce the risk of gout, while GLP-1 mimetics had only a neutral effect [4]. Furthermore, SGLT2 inhibitors are frequently prescribed alongside thiazide diuretics, considered the cornerstone for antihypertensive medications in patients with DM [97]. The hyperuricemic effect of diuretics can increase the risk of gout; therefore, co-administration with SGLT2 inhibitors may have a protective effect, which deserves further study [98].

The exact mechanism of SGLT2 inhibitors remains unclear, but two possibilities have been ruled out, namely, direct effects on urate reabsorption transporters or direct inhibition of urate transport via SGLT2 [48]. Instead, SGLT2 is likely to increase urinary glucose levels (glycosuria), thereby altering urate transport in the kidney. One key mechanism involves glucose transporter 9 (GLUT9), found in the apical (GLUT9b) and basolateral (GLUT9a) membranes of the proximal renal tubule [48,99]. Elevated glucose concentration in the tubule lumen may *trans*-stimulate UA secretion via GLUT9b, as shown in vitro, thereby promoting uricosuria [9,48]. Additionally glucose appears to exert a *cis*-inhibitory effect on GLUT9b, which is also expressed in the collecting ducts [100], potentially further limiting UA reabsorption [48]. In addition to GLUT9, uricosuria associated with SGLT2 inhibition may also involve the urate transporter-1 (URAT-1), responsible for 90% of UA reabsorption [101,102]. While direct inhibition of URAT1 by SGLT2 (e.g., canagliflozin, luseogliflozin) has been ruled out [48], indirect effects may occur. Specifically, SGLT2 inhibition reduces blood glucose levels, which may lead to a reduction in endogenous insulin secretion. Insulin was previously shown to decrease UA excretion in humans [103]; therefore, lower insulin levels could relieve this suppression and enhance uricosuria through URAT1 [101]. However, further studies are needed to support this hypothesis. Overall, it is likely that SGLT2 inhibitors promote urate excretion via two glucose-mediated mechanisms involving GLUT9 and URAT1; however, the overall contribution of URAT1 to uricosuria was less pronounced than GLUT9 in vitro [101].

SGLT2 inhibitors are among the most well-tolerated glucose-lowering drugs, providing significant CV and renal benefits beyond glucose control [104]. They have been shown to improve CKD, atherosclerotic heart disease, HF, blood pressure, body weight, and fat distribution [104,105]. However, they are associated with increased risk of genitourinary infections in women and individuals predisposed to such infections (e.g., those with a history of urinary tract infections, poor hygiene, or post-menopause) [9]. A meta-analysis found that luseogliflozin and dapagliflozin ranked highest in SU-lowering efficacy among the SGLT2 inhibitors [40].

The suspected uricosuric mechanism of action of GLP-1RAs includes inhibition of Na^+^/H^+^-exchanger type 3 (NHE3) in the renal proximal tubule, thereby increasing natriuresis and alkalization of the urine, which can promote increased excretion of UA, thereby reducing SU levels. However, the evidence for this mechanism was established in vitro in animal models and needs to be studied in humans [60]. The cardioprotective effects of GLP-1 and its mimetics may stem from their anti-inflammatory and anti-atherosclerotic properties, as demonstrated both in animal models [103] and in human studies [104]. These anti-inflammatory effects extend beyond CV protection and have been observed in the gastrointestinal tract, where GLP-1 reduces cytokine production in intraepithelial lymphocytes [105]. However, these findings are predominantly based on preclinical studies, and further research in humans is required.

## 9. Conclusions

Previous studies have indicated that SGLT2 inhibitors and GLP-1 mimetics may reduce SU levels and lower the risk of gout flares. However, the degree of SU reduction and the modulation of gout flare risk can vary among different drugs within these classes. This review examined approved SGLT2 inhibitors and GLP-1 mimetics and those currently in development, focusing on their chemical properties, pharmacological profiles, and potential therapeutic applications in managing gout.

## Figures and Tables

**Table 1:** Clinical data summary for SGLT2 inhibitors.

Name of Drug	Gout Incidence	SUA	Study Design	Participants	Study Duration	References
**Canagliflozin**		Reduced	Post hoc analysis of four phase III studies	T2DM patients (n = 115)	100 or 300 mg/day administered for 26 weeks	[25]
		Reduced	Post hoc analysis of the CREDENCE study	T2DM patients with kidney disease (n = 153, 224 in the canagliflozin and placebo respectively)	100 mg administered for 52 weeks	[26]
		Reduced	Post hoc analysis of CANVAS AND CANVAS-Renal studies	T2DM patients elevated CVD risk (n = 10,142)	100 or 300 mg/day up to 7 years (CANVAS) and 3.6 years (CANVAS-R)	[27]
**Empagliflozin**	Reduced gout flares	Reduced	A randomized, double-blind, placebo-controlled study. EMPA-REG OUTCOME trial.	T2DM patients with established atherosclerotic CVD (n = 7020)	10 and 25 mg/day up to 3 years.	[32]
	Reduced gout flares, gouty arthritis, and initiation of gout therapy	Reduced	A phase III randomized, double-blind parallel-group, placebo-controlled trial (EMPEROR-Reduced trial)	T2DM patients with heart failure (n = 3676)	10 mg/day. 4–100 weeks	[34]
		Reduced	A randomized prospective intervention study comparing liraglutide with empagliflozin	T2DM patients (25% with established CVD disease) (n = 62)	25 mg/day for 3 months	[35]
	Reduced acute gout, gouty arthritis, and initiation of gout therapy	Reduced	Post hoc analysis of EMPEROR-Preserved (a phase III double-blind, parallel-group, placebo-controlled, event-driven trial)	chronic heart failure patients with preserved ejection fraction (n = 5924)	10 mg/day up to 172 weeks	[36,37]
	Did not reduce the risk of gout	Reduced	Post hoc analysis of EMPA-KIDNEY (a phase III double-blind, parallel-group, placebo-controlled trial)	Patients with chronic kidney disease (n = 6609)	10 mg/day up to 18 months	[38]
**Dapagliflozin**	Reduced initiation of gout therapy. No information about gout flares	Reduced	Follow-up on the DAPA-HF study (phase III, placebo-controlled, randomized)	Patients with heart failure and reduced ejection fraction with and without T2DM (n = 3119)	10 mg/day for 52 weeks	[43]
	Reduced initiation of gout therapy and colchicine therapy		Post hoc analysis of DAPA-HF and DELIVER trials			[44]
		Reduced	Quartz study (randomized, placebo-controlled crossover study)	Adults with asymptomatic hyperuricemia (n = 36)	combination of verinurad (9 mg/day) and febuxostat (80 mg/day) with and without Dapagliflozin (10 mg/day) for 7 days	[45]
**Luseogliflozin**		Reduced	Single- and multiple-dose trial	Healthy subjects (n = 57, 24 for single and multiple doses respectively)	single dose (1–25 mg/day) and 7-day multiple dose	[48]
		Reduced	Phase II randomized, placebo-controlled, double-blind study	T2DM patients (n = 232)	0.5, 2.5 and 5 mg/day for 12 weeks	[49]
		Reduced	Phase III randomized, double-blind, placebo-controlled, parallel-group comparative study	T2DM patients (n = 148)	2.5 mg/day for 24 weeks	[50]
		Reduced	Pooled analysis of four phase III studies	T2DM with different renal impairment or BMI	2.5 mg/day (or up to 5 mg/day) for 52 weeks	[51]
**Ipragliflozin**		No change from baseline	Phase II double-blind, multicenter, placebo-controlled dose-response study	T2DM Japanese patients (n = 360)	12.5, 25, 50 or 100 mg/day) for 12 weeks	[53]
		Reduced	ASSIGN-K	T2DM patients (n = 367)	50 mg/day for 24 weeks	[54]
		Reduced	Randomized, open-label, active-controlled small trial	Inadequately controlled T2DM patients (n = 30)	50 mg/day for 12 weeks	[55]
**Tofogliflozin**		Reduced	Phase IV multicenter double-blind, placebo-controlled trial	Inadequately controlled T2DM patients (n = 211)	20 mg/day for 16 weeks (and 36 weeks open label extension)	[56]
		Reduced	Combined phase II and III randomized, placebo-controlled, double-blind, multicenter, parallel-group study	T2DM patients (n = 229)	10, 20 and 40 mg/day for 24 weeks	[57]
**Ertugliflozin**	Reduced incidence of gout events	Reduced	VERTIS CV—phase III, multicenter, double-blind, placebo-controlled	T2DM patients with atherosclerotic and cardiovascular disease (n = 8246)	5 and 15 mg/day up to 260 weeks	[59]

**Table 2:** Clinical data summary for GLP-1RA.

	SUA	Clinical Data	Participants	Design	References
**Exenatide**	Increased but also increased absolute UA excretion	Post hoc analysis	Healthy overweight subjects (n = 9)	Acute infusion of 10 µg exenatide for 150 min following 90-min placebo infusion	[63,64]
	No change	Acute randomized, double-blind, placebo-controlled, parallel-group study	T2DM patients (n = 52)	10 µg infusion	[64,65]
	No change	Prospective randomized clinical study	Obese individuals with T2DM (n = 44)	Obese individuals with T2DM receive 5 µg exenatide twice daily for 4 weeks followed by 10 μg twice daily until week 26	[67]
**Dulaglutide**	Reduced	Open observational study	Patients with T2DM (n = 20) previously treated with empagliflozin (10 mg/day)	Dulaglutide administration at 1.5 mg/week for 3–6 months	[70]
	No change	Open-label, parallel-group, randomized, controlled study	Patients with T2DM and non-alcoholic fatty liver disease (n = 64)	24-week administration of dulaglutide (0.75 mg/week for 4 weeks, 1.5 mg/week for 20 weeks)	[71]
	No change	Single-center, open-label, pilot study	T2DM patients previously treated with 50 mg/day sitagliptin (n = 40)	24-week dulaglutide administration (0.75 mg/week)	[72]
**Semaglutide**	Reduced	Retrospective cohort study	T2DM patients (n = 50)	Semaglutide was administered 0.25 mg/week and increased to 0.5 or 1 mg/week after 4 weeks for 3–6 months	[76]
	Reduced	Retrospective study	Chinese participants with obesity (n = 43)	Semaglutide administered at 0.25 mg/week increased every two weeks up to 1.0 mg/week for 24 weeks	[77]
	No change	Prospective clinical trial comparing semaglutide and empagliflozide	T2DM patients (n = 20)	Three-month administration of semaglutide (0.25 mg/week s.c. increase to 0.5 mg/week on week 5 and to 1 mg/week on week 9)	[78]
**Liraglutide**	Reduced	Retrospective study	Non-diabetic obese patients with metabolic syndrome (n = 46)	12-week administration of liraglutode 0.6 or 1.2 mg/day	[81]
	Reduced	Retrospective study	T2DM obese patients (n = 54)	Not mentioned	[82]
	No change	Longitudinal study comparing liraglutide vs. sitagliptin and linagliptin	T2DM patients with renal impairment (n = 139)	Liraglutide (0.9 mg/day) vs. sitagliptin (50 mg/day) and linagliptin (5 mg/day) administered for 48 months	[83]
	Reduced less than dapagliflozin and empagliflozin	Comparison study between liraglutide (0.9 mg/day) and dapagliflozin (5 mg/day) or empagliflozin (10 mg/day)	T2DM patients with renal impairment (n = 188)	Liraglutide administered 0.9 mg/day for 36 months	[84]
	No change	Open-label, parallel-group, randomized, controlled trial comparing liraglutide to empagliflozin combined with insulin therapy	T2DM patients naive to GLP-1RA or DPP4i treatment	24-week liraglutide (0.9 mg/day) vs. empagliflozin (10 mg/day) administration	[85]
	Reduced	Prospective, multicenter, observational study	T2DM patients (n = 151)	Liraglutide administration starting at 0.3 mg/day and increased to 0.9 mg/day for 12 weeks	[86]
	No change	Non-randomized, controlled, interventional study	T2DM obese patients (n = 15)	Liraglutide (0.6 mg/day titrated to 1.2 mg/day after 2 weeks) administered for 3 months	[87]
	No change	Parallel-group, randomized, double-blind, placebo-controlled study	T2DM patients (n = 62)	Liraglutide (0.6 mg/day titrated to 1.2 mg/day after 1 weeks) administered for 5 weeks	[88]
	Reduced	Randomized, double-blind, placebo-controlled, parallel-group study (LOSE-IT)	Obese individuals with knee osteoarthritis	8-week intensive Cambridge weight management plan + 52 weeks liraglutide (3 mg/day)	[89]
**Mazdutide**	Reduced	Randomized, double-blind placebo-controlled trial (GLORY-1)	Overweight or obese Chinese adults (n = 610)	Mazdutide administered at 4 mg/week and 6 mg/week vs. placebo for 48 weeks	[92]
	Reduced	Randomized, double-blind, placebo-controlled phase 2 study	Overweight or obese Chinese adults (n = 248)	Mazdutide administered at 3, 4.5, and 6 mg/week for 24 weeks	[90]
	Reduced	Randomized, placebo-controlled, dose-escalation, multiple ascending dose phase 1b study	Overweight or obese Chinese adults (n = 12)	Mazdutide administered at 3, 4.5 and 6 mg/week for 12 weeks	[93]
	Reduced	Randomized, placebo-controlled, multiple ascending dose, phase 1b study	Overweight or obese Chinese adults (n = 24)	Mazdutide administered for 12 weeks at 9 mg/week target dose and 16 weeks at 10 mg/week target dose	[91]
